# Enriched Environment Experience Overcomes Learning Deficits and Depressive-Like Behavior Induced by Juvenile Stress

**DOI:** 10.1371/journal.pone.0004329

**Published:** 2009-01-30

**Authors:** Yana Ilin, Gal Richter-Levin

**Affiliations:** Department of Psychology, The Institute for the Study of Affective Neuroscience (ISAN), University of Haifa, Mount Carmel, Haifa, Israel; James Cook University, Australia

## Abstract

Mood disorders affect the lives and functioning of millions each year. Epidemiological studies indicate that childhood trauma is predominantly associated with higher rates of both mood and anxiety disorders. Exposure of rats to stress during juvenility (JS) (27–29 days of age) has comparable effects and was suggested as a model of induced predisposition for these disorders. The importance of the environment in the regulation of brain, behavior and physiology has long been recognized in biological, social and medical sciences. Here, we studied the effects of JS on emotional and cognitive aspects of depressive-like behavior in adulthood, on Hypothalamic-Pituitary-Adrenal (HPA) axis reactivity and on the expression of cell adhesion molecule L1 (L1-CAM). Furthermore, we combined it with the examination of potential reversibility by enriched environment (EE) of JS – induced disturbances of emotional and cognitive aspects of behavior in adulthood. Three groups were tested: *Juvenile Stress* –subjected to Juvenile stress; *Enriched Environment* – subjected to Juvenile stress and then, from day 30 on to EE; and *Naïves*. In adulthood, coping and stress responses were examined using the elevated plus-maze, open field, novel setting exploration and two way shuttle avoidance learning. We found that, JS rats showed anxiety- and depressive-like behaviors in adulthood, altered HPA axis activity and altered L1-CAM expression. Increased expression of L1-CAM was evident among JS rats in the basolateral amygdala (BLA) and Thalamus (TL). Furthermore, we found that EE could reverse most of the effects of Juvenile stress, both at the behavioral, endocrine and at the biochemical levels. The interaction between JS and EE resulted in an increased expression of L1-CAM in dorsal cornu ammonis (CA) area 1 (dCA1).

## Introduction

Mood disorders affect the lives and functioning of millions each year. A greater understanding of the neural circuits underlying mood in both normal and abnormal affective states has been identified as one of the critical needs in the field of mood disorders research [1].

Stress, particularly when uncontrollable, excessive and/or prolonged, can produce a myriad of emotional and cognitive alterations [2]–[4]. In some individuals, stress can eventually trigger or exacerbate mood disorders, among which depression and bipolar disorders appear to be particularly linked to aversive life experiences [5]. Chronic stress procedures are currently widely used in experimental animals (mainly rodents) to model depression [6]–[9].

Many of the hormones secreted during stress have been shown to affect learning and memory processes [3], [10], [11]. Thus, stress has been shown to affect synaptic plasticity [3], [12], particularly hippocampal plasticity, dendrite morphology, neurotoxicity and neurogenesis within the dentate gyrus [13], [14]. Stress diminishes hippocampal synaptic plasticity, producing morphological changes in dendritic development, and decreasing neurogenesis in the dentate granule cells. Stress effects on the hippocampal formation and on memory involve other neural structures (e.g., hypothalamus) and neuromodulators (norepinephrine and γ-aminobutric acid (GABA)) [12].

Also, numerous studies have demonstrated that early-life stressful experiences affect both acute and long-term development of neuroendocrine, cognitive and behavioral systems. Exposure to stress or trauma during early childhood may disturb the formation of functional brain pathways, in particular, of the limbic circuits [15]–[18].

Previous findings from our group indicate that an exposure of rats to a relatively brief stressful experience during juvenility (27–29 days of age) has profound and long-lasting behavioral effects [19], [20]. In addition, a short-term juvenile exposure to variable stressors produced two types of impaired avoidance learning reminiscent of symptoms of both mood and anxiety disorders [20], [21].

The importance of the environment in the regulation of brain, behavior and physiology has long been recognized in biological, social and medical sciences [22]. Animals maintained under enriched conditions (EE) have clearly been shown to have reduced aggression [23], reduction of anxiety, fear and excitability [24]–[27], reduction of stress [28]–[30], brain function [31], [32] and better learning abilities [33] than those maintained under standard conditions. However, most of these studies have been carried out in animals with no history of early insults.

Several studies have demonstrated that cell adhesion molecules (CAMs) are involved in corticosterone (CORT) actions in memory and neuroplasticity. Relevant to the current study, the expression levels of a member of the immunoglobulin superfamily, L1-CAM, are considered to be regulated through glucocorticoid-mediated pathways [34]. Moreover, L1-CAM, has been implicated not only in cell interactions during nervous system development, but also in synaptic plasticity and memory formation in the adult brain [35]–[42]. During early development it promotes neurite outgrowth and fasciculation [43], axon pathfinding [44] and myelination [45].

Recent clinical and preclinical work has highlighted L1-CAM as particularly susceptible to showing alterations in stress-related disorders and depression [46]–[50].

Here, we studied the effects of stress during juvenility (JS) (27–29 days of age) on emotional and cognitive aspects of depressive-like behavior in adulthood, on HPA axis reactivity and on the expression of L1-CAM. Furthermore, we combined it with the examination of potential reversibility by enriched environment of JS – induced disturbances of emotional and cognitive aspects of behavior in adulthood.

Alterations in expression level of L1-CAM was checked in the prefrontal cortex (PFC), basolateral amygdala (BLA), dorsal cornu ammonis (CA) area 1 (dCA1) and thalamus (TL). This areas were chosen because they share extensive anatomic connections [51] and found to be affected by early life stress [52]–[54].

The exposure to JS resulted in both mood and anxiety symptoms. Furthermore, EE could reverse most of the effects of JS, at the behavioral, endocrine and at the biochemical levels.

## Results

### Body weight

**Figure 1:**
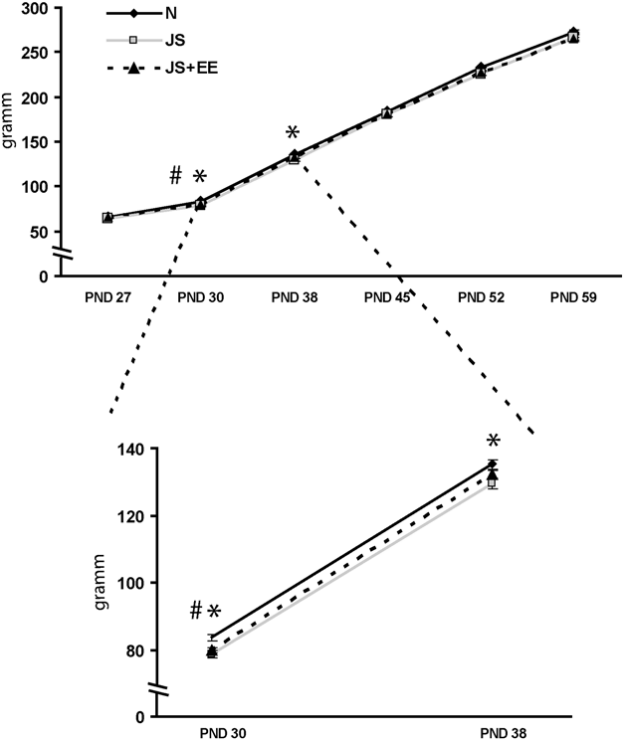
Body weight. JS (n = 39) and JS+EE (n = 39) exhibited less body weight gain when examined 24 h after the exposure to stress compared to Naïve group (n = 48). However, one week later (38 PND) this difference was observed only for the JS group. There was no difference between Naïve and JS+EE groups. Later on during the maturation process (at 45, 52, and 59 PND), this difference was no longer evident. *JS significantly different from Naïve (p<0.05); #JS+EE significantly different from Naïve (p<0.05).

Significant differences (p<0.05) were observed between the body weight gain of the three groups (Naïve, JS and JS+EE). Repeated measure analysis for lingering body weight gain revealed a significant main effect for each measure [WL = 0.005; F(1,174) = 17355.34; p<0.001] and for groups [F(2,114) = 3.84; p<0.024]. Post-hoc Tukey analysis at 30 post natal day (PND) indicated that in comparison with Naïve (unexposed) rats, juvenile-stressed rats (from both groups JS and JS+EE) exhibited less body weight gain when examined 24 h after the exposure to stress. However, one week later (38 PND) this difference was observed only for JS group, there was no difference between Naïve and JS+EE groups. However, later on during the maturation process (at 45, 52, and 59 PND), this difference was no more evident. These results indicate that though the stressor affected body weight gain in the short run, in the long run juvenile-stressed rats (from both groups JS and JS+EE) continued to develop normally in terms of their body weight gain (Figure 1).

### Behavioral Assessments in Adulthood

Animals were tested in the open field (OF) and elevated plus-maze (EPM) at 60 PND, after 1 month in different housing environments. Significant differences in behavioral parameters (activity and anxiety-like behavior) were observed. At the next, 61 PND, day animals were subjected to the TWS avoidance task. Learning abilities of the animals were also affected by the manipulations.

### Open Field

**Figure 2:**
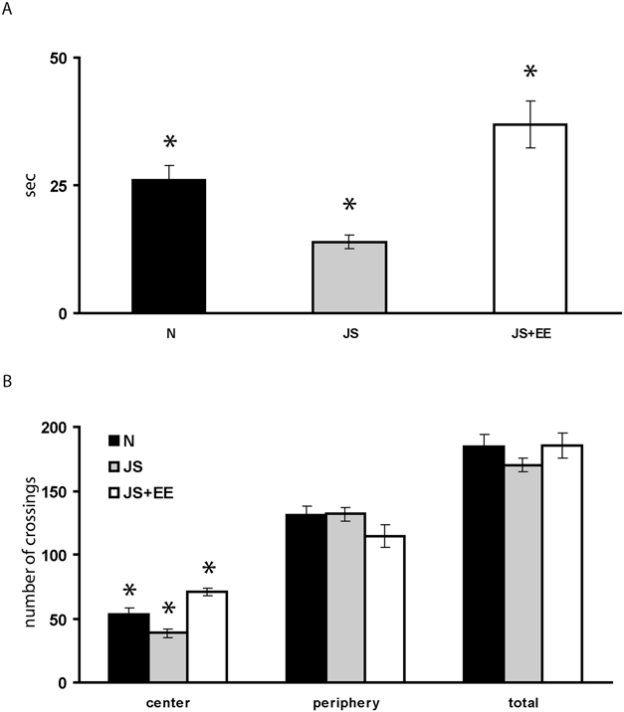
Open Field (OF) Test. (A) *Time spent in the open arena*. Time spent in the open arena of the OF of the JS (n = 17) group was significantly shorter than that of the Naïve (n = 20) and JS+EE (n = 17) groups. Time spent in the open arena of the JS+EE group was significantly longer than that of the Naïves and JS. Time spent in the open arena of the Naïve group was significantly longer than that of the JS, while being significantly shorter than that of the JS+EE group. (B) *The locomotor activity in the OF*. The number of center square crossing of the JS group was significantly lower than that of the Naïve and JS+EE groups. The number of center square crossing of the JS+EE group was significantly higher than that of the Naïves and JS. The number of center square crossing of the Naïve group was significantly higher than that of the JS, while being significantly lower than that of the JS+EE group. There was no difference between the groups in the number of periphery square crossing and total locomotor activity (total number of squares crossed) in the OF. *significantly different from all other groups (p<0.05).

One-way ANOVA revealed a significant effect of group on time spent in the open arena of the OF [F(2,51) = 12.75, p<0.001]. Post-hoc Tukey testing indicated that the time spent in the open arena of the OF of the JS group was significantly lower than that of the Naïve and JS+EE groups. The time spent in the open arena of the JS+EE group was significantly higher than that of the Naïves and JS. The time spent in the open arena of the Naïve group was significantly higher than that of the JS, while being significantly lower than that of the JS+EE group (Figure 2A).

One-way ANOVA revealed a significant effect of group on the number of center square crossing [F(2,51) = 16.54, p<0.001]. Post-hoc Tukey testing indicated that the number of center square crossing of the JS group was significantly lower than that of the Naïve and JS+EE groups. The number of center square crossing of the JS+EE group was significantly higher than that of the Naïves and JS. The number of center square crossing of the Naïve group was significantly higher than that of the JS, while being significantly lower than that of the JS+EE group (Figure 2B).

One-way ANOVA for the number of periphery square crossing showed no significant effect for groups [F(2,51) = 1.87, N.S.] (Figure 2B).

One-way ANOVA for the locomotor activity (total number of squares crossed) showed no significant effect for groups [F(2,51) = 0.92, N.S.] (Figure 2B).

### Elevated Plus Maze

**Figure 3:**
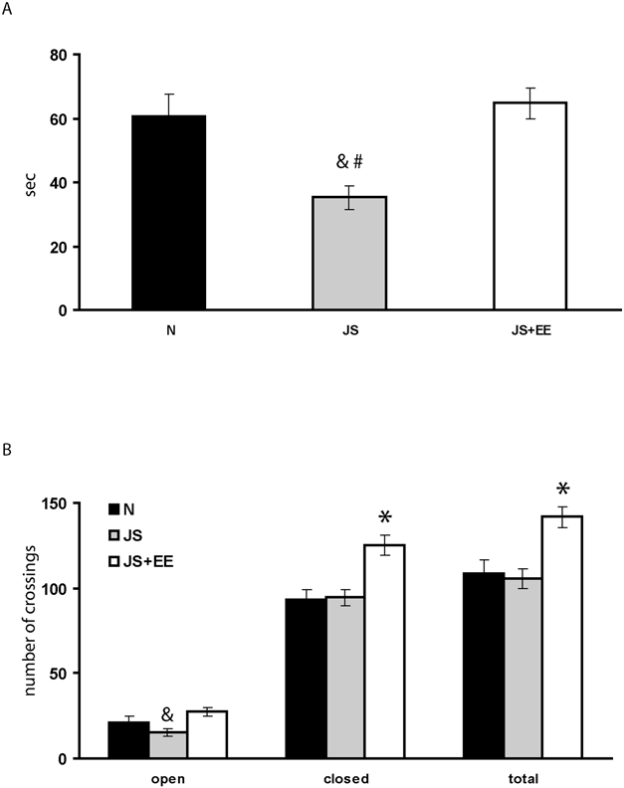
Elevated Plus Maze (EPM). (A) *Time spent in the open arms*. Time spent in the open arms of the JS (n = 29) group was significantly shorter than that of the Naïve (n = 31) and JS+EE (n = 30) groups. There was no significant difference between JS+EE and Naïve groups. (B) *The locomotor activity in the EPM*. The line crossing in the open arms of the JS group was significantly lower than that of the JS+EE group. Line crossing in the closed arms of the JS+EE group was significantly higher than that of the Naïve and JS groups. Total line crossing of the JS+EE group was significantly higher than that of the Naïve and JS groups. *significantly different from all other groups (p<0.05); &significantly different from JS+EE group (p<0.05); #significantly different from Naïve group (p<0.05).

One-way ANOVA revealed a significant effect of group on time spent in the open arms of the EPM [F(2,87) = 8.82, p<0.001]. Post-hoc Tukey testing indicated that the time spent in the open arms of the JS group was significantly lower than that of the Naïve and JS+EE groups. There was no significant difference between JS+EE and Naïve groups (Figure 3A).

One-way ANOVA revealed a significant effect of group on line crossing in the open arms of the EPM [F(2,87) = 4.32, p<0.016]. Post-hoc Tukey testing indicated that the line crossing in the open arms of the JS group was significantly lower than that of the JS+EE group (Figure 3B).

One-way ANOVA revealed a significant effect of group on line crossing in the closed arms of the EPM [F(2,87) = 10.07, p<0.001]. Post-hoc Tukey testing indicated that the line crossing in the closed arms of the JS+EE group was significantly higher than that of the Naïve and JS groups (Figure 3B).

One-way ANOVA revealed a significant effect of group on total line crossing in the EPM [F(2,87) = 8.89, p<0.001]. Post-hoc Tukey testing indicated that the total line crossing of the JS+EE group was significantly higher than that of the Naïve and JS groups (Figure 3B).

### Novel-setting exploration

**Figure 4:**
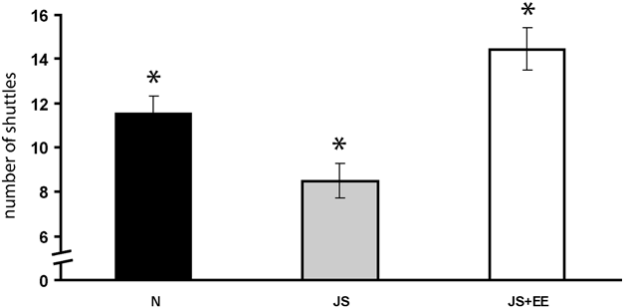
Exploration of a novel setting. Exploratory behavior of the JS (n = 38) group was significantly lower than that of the Naïve (n = 41) and JS+EE (n = 40) groups. The exploratory behavior of the JS+EE group was significantly higher than that of the Naïves and JS. The exploratory behavior of the Naïve group was significantly higher than that of the JS, while being significantly lower than that of the JS+EE group. *significantly different from all other groups (p<0.05).

One-way ANOVA revealed a significant effect of group on novel setting exploration [F(2,116) = 12.32, p<0.001]. Post-hoc Tukey testing indicated that the exploratory behavior of the JS group was significantly lower than that of the Naïve and JS+EE groups. The exploratory behavior of the JS+EE group was significantly higher than that of the Naïves and JS. The exploratory behavior of the Naïve group was significantly higher than that of the JS, while being significantly lower than that of the JS+EE group (Figure 4).

### Two-way shuttle (TWS) avoidance task

#### Avoidance responses

**Figure 5:**
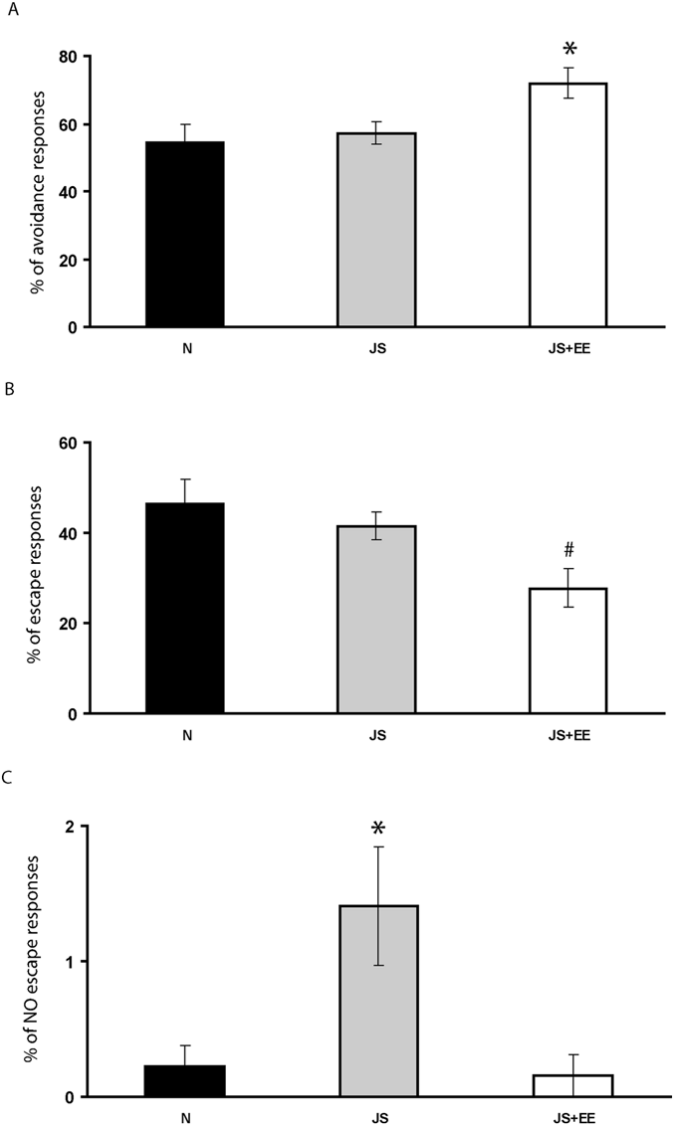
Two-Way Shuttle (TWS) Avoidance learning. (A) *Avoidance responses*. Percent of avoidance responses of the JS+EE (n = 8) group was significantly higher than that of the Naïve (n = 10) and JS (n = 8) groups. (B) *Escape responses*. Percent of escape responses of the JS+EE group was significantly lower than that of the Naïve and JS groups. (C) *No Escape responses*. Percent of no escape responses of the JS group was significantly higher than that of the Naïve and JS+EE groups. *significantly different from all other groups (p<0.05); #significantly different from Naïve group (p<0.05).

One-way ANOVA revealed a significant effect of group on percent of avoidance responses during TWS avoidance task [F(2,24) = 3.79, p<0.037]. Post-hoc Tukey testing indicated that percent of avoidance responses of the JS+EE group was significantly higher than that of the Naïve and JS groups (Figure 5A).

#### Escape responses

One-way ANOVA revealed a significant effect of group on percent of escape responses during TWS avoidance task [F(2,24) = 3.96, p<0.033]. Post-hoc Tukey testing indicated that percent of escape responses of the JS+EE group was significantly lower than that of the Naïve and JS groups (Figure 5B).

#### No Escape responses

One-way ANOVA revealed a significant effect of group on percent of no escape responses during TWS avoidance task [F(2,24) = 6.75, p<0.005]. Post-hoc Tukey testing indicated that percent of no escape responses of the JS group was significantly higher than that of the Naïve and JS+EE groups (Figure 5C).

### Endocrine and Molecular Assessments in Adulthood

At PND 60, between 10:00 and 12:00 h, Naïve, JS and JS+EE groups of animals, without previous history of testing, were taken directly from their home-cages for brain and trunk blood collection.

### Concentrations of corticosterone

**Figure 6:**
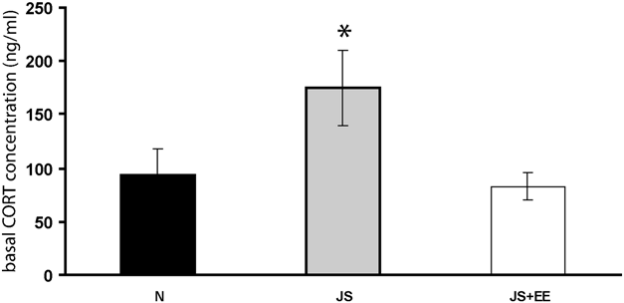
Serum corticosterone concentration. Basal CORT concentration of the JS (n = 9) group was significantly higher than that of the Naïve (n = 10) and JS+EE (n = 8) groups. *significantly different from all other groups (p<0.05).

One-way ANOVA revealed a significant effect of group on basal CORT concentration [F(2,23) = 6.14, p<0.007]. Post-hoc Tukey testing indicated basal CORT concentration of the JS group was significantly higher than that of the Naïve and JS+EE groups (Figure 6).

### L1-CAM expression

**Figure 7:**
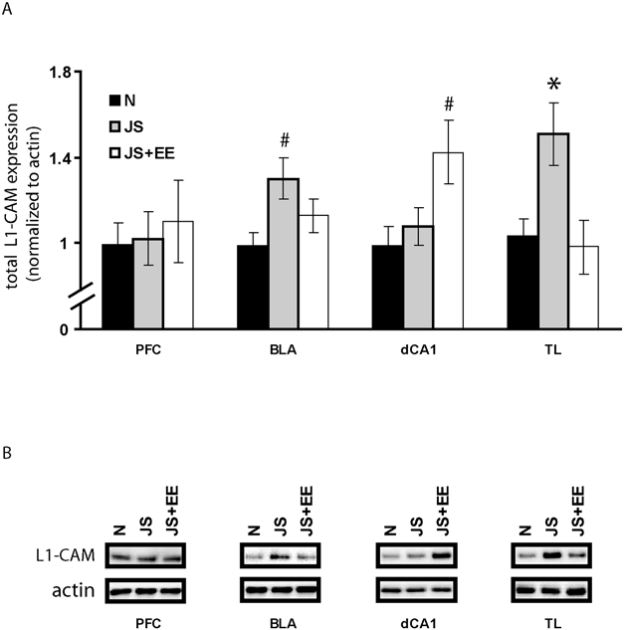
L1-CAM expression at post natal day 60. (A) L1-CAM expression was measured at 60 PND in the PFC, BLA, dCA1 and TL (Naïve (n = 10); JS+EE (n = 8); JS+EE (n = 8)). Expression levels are depicted as the ratio between the total L1-CAM expression level and β-actin levels in each brain area (i.e. L1-CAM/β-actin), normalized to the Naïve group. In the PFC: no difference between the groups for L1-CAM expression levels. In the BLA: L1-CAM expression levels of the JS group was significantly higher than that of the Naïve group. In the dCA1: L1-CAM expression levels of the JS+EE group was significantly higher than that of the Naïve group. In the TL: L1-CAM expression levels of the JS group was significantly higher than that of the Naïve and JS+EE groups. (B) L1-CAM representative immunoblots. Bottom rows: β-actin; Top Rows: L1-CAM. *significantly different from all other groups (p<0.05); #significantly different from Naïve group (p<0.05).

L1-CAM expression was measured at 60 PND in the prefrontal cortex (PFC), basolateral amygdala (BLA), dorsal cornu ammonis (CA) area 1 (dCA1) and thalamus (TL) (Figure 7). Expression levels are depicted as the ratio between the total L1-CAM expression level and β-actin levels in each brain area (i.e. L1-CAM/β-actin), normalized to the Naïve group.

#### In the PFC

One-way ANOVA for L1-CAM expression levels in the PFC showed no significant effect for the group [F(2,26) = 0.17, N.S.].

#### In the BLA

One-way ANOVA for L1-CAM expression levels in the BLA revealed significant effect for the group [F(2,23) = 4.22, p<0.027]. Post-hoc Tukey testing indicated that L1-CAM expression levels of the JS group was significantly higher than that of the Naïve group.

#### In the dCA1

One-way ANOVA for L1-CAM expression levels in the dCA1 revealed significant effect for the group [F(2,23) = 4.30, p<0.026]. Post-hoc Tukey testing indicated that L1-CAM expression levels of the JS+EE group was significantly higher than that of the Naïve group.

#### In the TL

One-way ANOVA for L1-CAM expression levels in the TL revealed significant effect for the group [F(2,25) = 6.09, p<0.007]. Post-hoc Tukey testing indicated that L1-CAM expression levels of the JS group was significantly higher than that of the Naïve and JS+EE groups.

## Discussion

Social/Environmental stress in early life such as maternal separation, isolation, poverty, etc. is not avoidable in many children. Cognitive deficits progressively emerging with development are the results of complex interactions between genetic and environmental factors [55]–[57], and evidence suggests that EE experience can attenuate or reverse a variety of cognitive deficits [58].

This study was designed to experimentally investigate the effects of EE during adolescents on JS rats. We found that, JS rats showed anxiety- and depressive-like behaviors in adulthood, altered HPA axis activity and L1-CAM expression pattern through limbic system areas and the thalamus. Furthermore, we found that EE could reverse most of the effects of JS, both at the behavioral, endocrine and at the biochemical levels.

### Behavioral Assessments in Adulthood

Our JS protocol resulted in a variety of behavioral changes in the rodents that might be regarded as behavioral correlates of depressive-like symptoms in humans. In our experiment rats were compared in the following tests: (1) OF and EPM tests for the assessment of anxiety level as one of the possible components of depressive state [24], [59]; (2) novel-setting exploration as motivational/hedonic state measure [60]–[62]; (3) TWS avoidance task for revealing a possible sign for cognitive disturbances or learned helplessness (LH) behavior, as an analogue of impaired coping state in depression [63], [64].

Exposure to the JS transiently delayed body weight gain. In comparison with Naïve rats, both juvenile-stressed rats (JS and JS+EE) exhibited less body weight gain when examined 24 h after the exposure (at 30 PND), indicating that both groups were affected in the same way by the stressors. However, one week later, 37 PND, JS rats continued to show less body weight gain than Naïve animals, while JS+EE rats were no longer different from Naïves. This finding indicates that the EE protocol started to have an impact already from the first week.

However, later, during the maturation process, the body weight gain difference was no longer evident, indicating that our variable stress procedure affected body weight gain in the short run; in the long run both JS groups (JS and JS+EE) continued to develop normally in terms of their body weight gain. The same pattern of weight changes was found also by Brunson et. al. [65].

The emotional consequence of exposure to JS was examined in both the OF and the EPM. High anxiety levels of the JS group were found in both tests. In the OF there was a decrease in the time spent in the central arena and center square crossing by JS rats compared to Naïve and JS+EE rats. These findings confirm previous findings of high anxiety level of JS rats even 1 month after the exposure to the stress protocol [19], [20]. EE not only reversed high anxiety levels of JS, but reduced them even below the levels of Naïve animals.

Similar results were obtained in the EPM. JS reduced time spent in open arms (as indicated both by time in open arms and open arms crossing). Exposure of animals to the EE condition during 1 month after JS completely reversed this effect. In fact, EE in JS animals increased total exploratory behavior even beyond that of Naïve animals.

The initial activity of a rat placed in a novel surrounding (e.g. novel setting exploration) can be taken as an indicator of its emotional and motivational state [60]. It is assumed that a novel context/situation reflects both the stress and the rewarding component of novelty. It has been proposed that reduced sensitivity to rewards in rodents might be homologous to human anhedonia [66]. In rats, decreased exploratory activity in a novel environment might reflect decreased motivation or drive, a behavior representing “refractory loss of interest” [60], [67] and may also be related to an hedonic deficit, since novelty is rewarding [61], [62]. In our model JS rats exhibited reduced novel-setting exploration compared to Naïve and JS+EE rats. This reduced exploratory activity may represent the loss of interest in new stimulating situations and may imply the presence of motivational deficits. In contrast, novel-setting exploration of the JS+EE rats not only was higher from JS rats but was also higher from Naïve rats.

During TWS avoidance task JS rats were not different from Naïve-controls in the total number of avoidance or escape responses, but showed significantly more No Escape responses. The increased rates of escape failure (no escape responses) during this task that we found among JS rats may also imply an emotional disruption. Such increases in escape failures were suggested to correspond to learned helplessness, representing, in animals, depressive symptoms of non-responsiveness [68]. EE completely reversed this effect. Furthermore, EE increased total number of avoidance responses even beyond that of Naïve-controls. Improved learning and memory by EE is one of the most consistent findings in the literature [69], [70]. The present results confirm this finding and extend its validity by showing that this effect even overcomes the effects of JS.

Furthermore, exposure to stressors during juvenility affected the HPA axis baseline activity. Analysis of basal circulating CORT levels revealed elevated levels in the JS group, as compared to Naïve and JS+EE groups. Serum corticosterone was used as the traditional anxiety/stress marker [71]. This result provides independent support to indicate that the JS group indeed experienced significantly higher levels of anxiety than either of the other groups. This finding is in agreement with reported physiological abnormality of resting level titers of the hormone in depressed humans [68], [72], [73]. EE reversed also this effect of JS.

Overall, JS appears to trigger anxiety- and depressive-like behaviors; EE was found to be able to reverse these effects. Moreover, EE not only reversed most of JS-induced disruptions but rather, in some parameters made the animals less anxious, more motivated and with better learning abilities compared also with Naïve animals.

L1-CAM, together with other members of the L1 subfamily, is critical for several early development processes like axon outgrowth, fasciculation, neuronal migration and survival [46], [74]–[76]. Furthermore, L1-CAM restriction throughout post-weaning and to adulthood developmental phase also affected stress responsiveness and cognitive functions in adulthood [77], suggesting a key role for L1-CAM in development related processes during adolescence.

In the current study, exposure to stressors during juvenility altered the expression levels of L1-CAM throughout the monitored brain regions. Increased expression of L1-CAM was evident among JS rats in the BLA and thalamus. In the thalamus, EE completely reversed this effect, while in the BLA it only reduced it.

Exposure to stressors during juvenility affected the HPA axis baseline activity as was indicated by elevated basal CORT levels. The amygdala shares extensive anatomic connections with the thalamus [51]. Both these areas serve as feedback sites of HPA regulation in stressed animals [78], so that alteration of L1-CAM expression by JS and by EE could be related to the alterations found in CORT levels under these conditions.

Individual variations in L1-CAM mRNA levels were positively correlated with plasma CORT concentrations and anxiety-like behaviors [38]. It was suggested that chronic-stress induced increased L1-CAM levels may contribute to the chronic stress-associated emotional and cognitive impairments [39], [80]. In addition, in the adult brain, L1-CAM regulation is affected by continuous increased CORT levels or chronic stress exposure [39]. Thus, elevated basal levels of CORT could explain the observed amygadalar and thalamic L1-CAM alterations.

Since L1-CAM was implicated in repair processes in the adult lesioned CNS [81]–[83], chronic-stress induced increased L1-CAM levels were suggested to represent the activation of a neuroprotective mechanism [39], [41], [84]. However, early life stress could disrupt the information processing in the cortex and thalamus of the developing brain, and limbic system particularly, of juvenile rats leading to cognitive and affective disorders. Controversially, the limbic system is most probably modified by EE [24]. Thus, EE experience could rescue the early life induced development disruptions by triggering the release of nerve growth factors, activating neurotransmitter receptors, or enhancing neurogenesis [69], [85], [86].

The interaction between JS and EE resulted in an increased expression of L1-CAM in dCA1, beyond that of Naïve-controls and JS rats. EE has been found to have profound and long-lasting neural and physiological consequences on the hippocampus. EE has been shown to induce higher hippocampal expression of glucocorticoid type II receptor mRNA [87]; enhanced hippocampal field potentials [88], [89]; and hippocampal neurogenesis in adult animals [32], [69]. Thus, L1-CAM increased levels in the dCA1 area could reflect neuroprotective mechanism and the neurogenesis that occurred through interaction between JS and EE.

EE was also found to improve the acquisition and long-term retention of a two-way active avoidance [70]. These changes could be correlated with the behavioral effects of EE compared to controls. It is thus tempting to suggest that these alterations in an area (CA1) associated with the behavior are relevant to the behavioral effects of EE. Further experiments are required to clarify this possibility

In conclusion, our data show that JS applied in rats induces a broad spectrum of behavioral changes reminiscent of depressive symptoms in humans. These results may be helpful for elucidating cellular and molecular mechanisms involved in cognitive deficits and affective disorders caused by early life stress. On the other hand, our findings suggest that EE may be useful to prevent these devastating effects in young adults following childhood stress.

## Methods

### Subjects

Male Sprague Dawley rats (SD), 22 days old, weighing 35–49 g were purchased from Harlan (Jerusalem, Israel) and habituated in the Brain and Behavior Research laboratory facilities for five days. Three animals were housed per cage in 75×55×15 cm Plexiglas cages in temperature-controlled (23±1°C) animal quarters on a 12∶12 light-dark cycle (lights on 07:00–19:00 hours). They had *ad libitum* access to standard Purina rodent chow pellets and water.

### Ethical approval

All procedures and tests were approved by the Institutional Animal Care Committee and adhered to the guidelines of the US Institute of Laboratory Animal Research's Guide for the Care and Use of Laboratory Animals.

### Three groups of SD rats were used

1. JS subjected to variable stress at post natal days (PND) 27–29
2. JS+EE subjected to variable stress at 27–29 PND and at 30 PND were transfered to EE housing conditions.
3. Naïve rats.

### Juvenile stress procedure

We have designed a juvenile short-term variable stressor protocol [20] in which rats were exposed to a different stressor every day for three days (see below). Stress exposure took place during juvenility (ages 27–29 days) at approximately midday (12:00–14:00) in designated experimental rooms (a different room each day) away from the *vivarium*.

- Day 1. (aged 27 d) Forced swim: 10 min forced swim in an opaque circular water tank (diameter 0.5 m; height: 0.5 m; water depth 0.4 m), water temperature 22±2°C (adapted from Avital et. al. [90]).
- Day 2. (aged 28 d) Elevated platform: three 30 min trials; ITI (Inter-Trial Interval): 60 min in the home cage. Elevated platform: 12×12 cm at a height of 70 cm above floor level, located in the middle of a small closet-like room (adapted from [91].
- Day 3 (aged 29 d) Restraint stress: Rats were placed in a metal mesh restraining box (11×5×4 cm) that prevented forward-backward movement and limited side-to-side mobility, but did not discomfort the animal in any other way. Rats remained in the restraining box for 2 hrs at 25°C under dim illumination.

Protocols were applied in parallel to rats in the stress groups, so as not to isolate any rat in its home cage. Upon completion of the each of the stress procedures, rats were returned to their home cage.

### Environmental Enrichment procedure

Enriched Environment was defined in terms of combination of physical environment and partially social housing conditions. Therefore, animals were housed in larger and higher cages provided with differently shaped plastic containers, colored platforms and suspended objects. The objects were changed twice a week. Once a week all animals from this group were taken together to another enriched box with different objects, wheel, one apple, carrot, cucumber and 50 g of granola.

For both housing conditions: standard and EE, the sawdust of the cage was changed once a week in association with measurement of animals body weight. Rats were put in standard and EE cages at the age 30 PND and maintained in their housing conditions throughout all the experimental assessment.

### Experimental design

In the present study in order to prevent the tests from influencing one another, different rats were used for each of the following experiments: (1) behavioral measurements; (2) corticosterone concentrations and L1-CAM expression.

### Behavioral Assessments in Adulthood

In adulthood, 60–61 PND, coping and stress responses were examined using the open field test, elevated plus-maze test, the novel-setting exploratory behavior, two-way shuttle (TWS) avoidance task.

#### Open field test (OF)

The apparatus is a quadrant box, 90 cm length with 30 cm wall, divided into 15×15 cm squares. Animal was placed in the center of the field and the following variables were recorded for 5 min: the number of squares crossed and center square entries. The open field was cleaned after each rat. The test room had a dim illumination (40 W) for decreasing the aversiveness of the test.

#### Elevated plus-maze test (EPM)

The apparatus is elevated 80 cm above a floor and exposed to dim illumination. It consists of two opposite open arms (45×10 cm) and two opposite closed arms of the same size with walls 10 cm high. The arms are connected by a central square (10×10 cm). Each rat was placed on the central-platform facing an open arm and was allowed to explore the maze for 5 min. Each test was videotaped and scored by an independent observer. Arm entry was defined as entering an arm with all four paws. The following terms were used: durations in open arms, open and closed arm crossing and total crossing of all arms.

#### Novel-setting exploration

Rats were placed in the two-way shuttle avoidance apparatus described below, although it was in an inoperative mode, and were allowed to explore both compartments for a total of 10 min. Crossingover between compartments provided an index of exploratory behavior.

#### Two-way shuttle (TWS) avoidance task

Immediately after the exploratory behavior assessment a training session began. Apparatus: The TWS box, placed in a dimly-lit, ventilated, sound-attenuated cupboard, is a rectangular chamber (60×26×28 cm) divided by an opaque partition with a small flap passage (10×8 cm) that connects two equal sized, side-by-side, cube-shaped compartments. Both metal grid floors of the compartments are weight sensitive and electrifiable. Micro-switches transmit information about the location of the rat to a computer control and data collection program. This program controls both conditioned stimulus (CS) presentations (a tone produced by loudspeakers located on the distal walls of the compartments) and unconditioned stimulus (US) – electric shock deliveries (to the animals' feet through the compartment floor, by a Solid State Shocker/Distributor, Coulbourn Instruments Inc. Lehigh Valley, PA, USA). The TWS avoidance task: One session comprises of 80 “trace conditioning” trials. CS: 10 s tone presentation; US: immediately following the termination of the CS an electric shock (1.2 mA) will be delivered for a maximum of 10 s; ITI: (randomly varying) 30±12 s. Rats could perform one of the following behaviors: (1) **Avoidance** - shuttling to the adjacent chamber of the apparatus while the tone was on, thus avoiding the shock altogether; (2) **Escape** - shuttling to the other compartment after the shock began, thus reducing exposure to the shock; (3) **No Escape** - not shuttling to the adjacent chamber, thus receiving the full length of the shock.

### Corticosterone (CORT) radioimmunoassay

Trunk blood was collected into plastic tubes following decapitation between 10:00 and 12:00 h. Samples were centrifuged at 3000 rpm for 20 min at 4°C. Approximately 1 ml of serum from each rat was collected into 1.5 ml Eppendorf tubes and stored at −80°C. The tubes were numbered, but not labeled, so that analysis of CORT levels was blind to the experimental procedure followed. CORT levels were assessed using DSL/10/81000 ELISA kits (DSL, Texas). The sensitivity of the CORT assay was 12.5 µg/L. Within-assay variation was less than 10% at 100 µg/L, and between-assay variation was less than 15% at 100 µg/L.

The CORT serum concentrations were used to further corroborate basal stress levels.

### Brain extraction

At the PND 60 animals were taken from their home cages and sacrificed, their brains was extracted, immediately frozen in isoproponol and stored at −80°C. Bilateral tissue punches with seventeen-gauge needle of prefrontal cortex (PFC), basolateral amygdala (BLA), dorsal cornu ammonis (CA) area 1 (dCA1) and thalamus (TL) were obtained from ∼1.5 mm coronal sections cut in a cryostat at −20°C. The coronal sections were approximately +4.0 (PFC), −1.8 (BLA) and −2.0 (dCA1 and TL) from bregma, respectively [92].

The tissues were immediately homogenized in an ice-cold glass/Teflon homogenizer (885502-0019; KONTES GLASS COMPANY, Vineland, NJ, USA) using 50 Teflon/glass mortar strokes in 300 µl of ice-cold NP-40 lysis buffer (20 mM Tris HCl, 20 mM EDTA, 1% NP-40, 137 mM NaCl, 10% glycerol, pH 8), with freshly added with the following protease inhibitors: 0.1 mM sodium orthovanadate, 1 µg/ml leupeptine, 1.6 µg/ml aprotinin and 5 mM NaF and 1 µg/ml protease inhibitor cocktail P2714 (from Sigma). 30 µl of each lysate were saved for further protein concentration by Bradford analysis. The regions were immediately homogenized with ice cold sodium dodecyl sulfate (SDS) sample buffer (20% glycerol, 10% β-mercaptoethanol and 20% SDS, 2.33 gr bromophenol blue in 62.5 mM Tris-HCl, pH 6.8) was added to each remaining lysate, thoroughly mixed and denatured 5 min at 95°C. The denatured proteins were stored at −80°C for further analysis.

### Immunoblot analysis

Protein concentration was monitored using Bradford assay, and equal amounts of loaded protein were verified using β-actin staining (1∶1000, II - α-Goat 1∶10000, BIOCHEM; 10% acrylamide). No differences were observed between the groups in β-actin concentrations in any of the examined regions. Individual samples from each region of each rat (20 µg) were loaded onto 7.5% SDS-PAGE gels. Following electrophoresis gels were transferred by wet transfer tanks to nitrocellulose membranes and stained against L1-CAM: (α-NCAM-L1-(C-20) Santa Cruz-SC-1508-1∶1000, II - α-Goat 1∶10000, BIOCHEM). The membranes were developed using the enhanced chemiluminescence light (ECL) (Amersham, Piscataway, NJ) reaction with a charge coupled device (CCD) camera (XRS BioRad).

### Quantification

Densitometric analysis of L1-CAM and β-actin immunoreactivity was conducted using Quantity One 1-D Analysis software. Each sample was measured relative to the background, and expression levels were calculated as the Optical Density (OD) ratio between the β-actin and L1-CAM of each sample.

The results were normalized to Naïve group values.

### Statistical Analysis

The results are expressed as means±SEM. For statistical analysis, a one-way ANOVA test was applied. For post-hoc comparisons, the Tukey contrast test was used with an α level of 0.05, unless otherwise noted.
